# Heat stress on sperm quality in dogs: effect of natural antioxidant supplementation

**DOI:** 10.3389/fvets.2025.1603458

**Published:** 2025-08-07

**Authors:** Lucrezia Forte, Matteo Burgio, Annalisa Rizzo, Aristide Maggiolino, Alice Carbonari, Pasquale De Palo, Giovanni Michele Lacalandra, Vincenzo Cicirelli

**Affiliations:** Department of Veterinary Medicine, University of Bari Aldo Moro, Bari, Italy

**Keywords:** thermal stress, dogs, antioxidants, semen quality, biochemical profile

## Abstract

**Introduction:**

Heat stress negatively affects canine reproductive health by inducing oxidative stress and impairing sperm quality. This study assessed the efficacy of a polyphenolic extract from Loblolly pine (*Pinus taeda*) lignin (PTHL), in mitigating heat stress-induced oxidative damage and preserving sperm quality in dogs.

**Methods:**

Forty clinically healthy male dogs were divided into two groups: a control (CON) group receiving a standard diet and a PTHL-supplemented group for 90 days. During summer, dogs were exposed to natural heat stress, and key parameters, including serum biochemical markers, oxidative stress indicators, antioxidant enzyme activity, and sperm quality were evaluated. Mixed-effects models analyzed group, time, and interaction effects.

**Results and discussion:**

PTHL supplementation significantly reduced plasma TBARS and seminal d-ROMs levels (*P* < 0.01), indicating lower lipid peroxidation and confirming its protective effects. Antioxidant capacity improved in the PTHL group, with increased activities of antioxidant enzyme activity (SOD, CAT, and GSPx) and elevated plasma Ferric Reducing Antioxidant Power (FRAP) levels (*P* < 0.01). Notably, the PTHL group demonstrated higher progressive motility (*P* < 0.01) and a greater percentage of rapid-movement sperm (*P* < 0.01) at 90 days, indicating improved sperm function under heat stress.

**Conclusion:**

These findings suggest that PTHL enhances antioxidant defenses, mitigating heat stress-induced reproductive impairment. This natural strategy may improve fertility in dogs facing environmental challenges. Future studies should explore different dosages and extended supplementation to optimize its benefits.

## 1 Introduction

Heat stress is a critical environmental factor affecting animal physiology and reproduction, particularly in mammals with limited thermoregulatory capacities, such as dogs (1). Elevated ambient temperatures disrupt homeostasis by inducing oxidative stress, altering metabolic pathways, and impairing organ function (2, 3). In male dogs, heat stress has been associated with a decline in sperm quality, compromised spermatogenesis, and reduced reproductive efficiency, leading to transient or even prolonged infertility (4). Understanding the mechanisms underlying these adverse effects and developing mitigation strategies is essential for preserving canine fertility, especially in breeding programs and working dogs exposed to challenging climatic conditions. In this context, the Thermo-Hygrometric Index (THI) is a valuable tool for assessing environmental heat load (5); in dogs, THI values above 72–74 are generally recognized as critical thresholds, beyond which physiological signs of heat stress begin to manifest (4). One of the primary pathways through which heat stress affects reproductive function is oxidative stress, characterized by excessive production of reactive oxygen species (ROS) overwhelming endogenous antioxidant defenses (6). ROS, including superoxide anions, hydrogen peroxide, and hydroxyl radicals, are natural byproducts of aerobic metabolism and play dual roles in biological systems (7). While they are involved in essential processes such as sperm capacitation and immune responses, their overproduction leads to lipid peroxidation, protein oxidation, and DNA fragmentation, ultimately impairing sperm function (2). Due to their high polyunsaturated fatty acid (PUFA) content and low intrinsic antioxidant capacity, spermatozoa are particularly vulnerable to oxidative damage, which can manifest as reduced motility, structural abnormalities, and apoptosis (8). Heat stress not only increases oxidative stress but also affects systemic physiology, leading to metabolic alterations that can further compromise reproductive function. Studies have shown that prolonged exposure to high temperatures reduces testosterone production, impairs Sertoli and Leydig cell function, and disrupts the spermatogenic cycle (9–14). Additionally, elevated temperatures promote peripheral vasodilation, leading to increased glucose uptake and subsequent hypoglycemia, which may exacerbate cellular stress responses (15). The combined impact of oxidative and metabolic stressors underscores the need for effective interventions aimed at preserving sperm integrity and function under heat stress conditions.

Dietary antioxidant supplementation has emerged as a promising strategy to counteract oxidative damage and support male reproductive health (16, 17). Antioxidants, particularly polyphenols, exhibit strong free radical-scavenging properties, modulating cellular redox balance and enhancing enzymatic antioxidant defenses (18). Among these, lignin-derived polyphenolic compounds from Loblolly pine (*Pinus taeda*) have gained interest due to their ability to improve antioxidant status and mitigate oxidative damage in various physiological contexts (17, 19–21).

The selection of *Pinus taeda* hydroxylated lignin (PTHL) for this study is based on previous findings from our group, in which dietary supplementation with this compound improved semen quality in healthy dogs under normal environmental conditions. Specifically, PTHL enhanced total antioxidant capacity and improved sperm parameters such as motility, concentration, and semen volume, likely through the upregulation of endogenous antioxidant enzymes such as superoxide dismutase, catalase, and glutathione peroxidase (17). Considering that heat stress has been shown to exert the opposite effect on these same parameters, reducing semen quality through oxidative and metabolic stress (17), we identified PTHL as a biologically plausible candidate for a targeted intervention. This rationale supports the hypothesis that PTHL may counterbalance the deleterious effects of elevated temperatures on male fertility. However, its protective effects under heat stress conditions remain unexplored.

This study aims to assess the efficacy of PTHL dietary supplementation in mitigating oxidative stress and preserving sperm quality in dogs subjected to heat stress. We hypothesize that PTHL supplementation enhances the antioxidant defense system, reduces oxidative damage, and improves semen characteristics compared to non-supplemented controls. By addressing this knowledge gap, our research provides novel insights into the role of dietary antioxidants in safeguarding reproductive function under adverse environmental conditions, with potential implications for canine breeding management and welfare.

## 2 Materials and methods

This prospective, randomized clinical study was conducted in compliance with the ethical guidelines of the Animal Welfare Committee. Approval was granted by the Institutional Review Board (approval number: 656/18 – III/13). Informed consent was obtained from all dog owners.

### 2.1 Animals

The study was conducted from May to September 2024 in southern Italy. A total of 40 male mixed-breed dogs, aged 2–4 years and weighing between 15 and 25 kg, participated in the trial. All dogs were owned, always kept outdoors, and housed individually. They were fed a standardized diet [10 g/kg of body weight (BW) daily, composition detailed in Table 1] twice a day, starting 30 days before the trial commenced. To ensure their health status, all dogs underwent a clinical examination 30 days before the start of the study. Each animal was subjected to both general and specialized reproductive tract assessments. Additionally, an ultrasound examination of the reproductive system was performed, and all dogs were confirmed to respond positively to sperm collection via digital manipulation. Exclusion criteria included obesity (weight exceeded its ideal weight by more than 30%) (22), white or long hair, and any medication use within the previous 30 days.

**Table 1:** Composition of diet commercial feed.

**Item**	**On 100 g of product**
Moisture	10 g
Raw protein	28 g
Fat	15 g
Raw fibers	3 g
Ashes	9 g
Vitamin A	1,500 IU
Vitamin D	100 IU
Vitamin E acetate (alpha-tocopherol 91%)	12.5 mg
Vitamin B2	2.6 mg
Vitamin B6 (pyridoxine hydrocloride)	0.5 mg
Vitamin B1 (thiamine mononitrate)	0.6 mg
Choline chloride	75 mg
Iodine (anhydrous calcium iodate)	0.075 mg
D-panthotenic acid	1 mg
Vitamin H (Biotin D)	0.05 mg
Calcium	0.5 g
Vitamin K3 (menadione)	0.125 mg
Vitamin PP (nicotine acid)	2.5 mg
Vitamin B12	0.0035 mg
Folic acid	0.1 mg
Cobalt (basic cobalt carbonate)	0.015 mg
Iron (ferrous carbonate)	2 mg
Manganese (manganous oxide)	4 mg
Copper (Copper sulfate, pentahydrate)	1 mg
Selenium (sodium selenite)	0.01 mg
Zinc (zinc oxide)	3 mg

### 2.2 Experimental protocol

The dogs were randomly assigned to one of two equal-sized groups (*n* = 20) using www.randomizer.org. One group served as the experimental group (PTHL), while the other functioned as the control group (CON). The PTHL group received a daily oral dose of 1 g per dog (about 50 mg/kg/day) of a supplement containing *Pinus taeda* hydrolyzed lignin (Oxyphenol^®^, I-Green, Padua, Italy) (Table 2) for the entire 120-day trial (4). The supplement was administered in powder form according to the manufacturer's guidelines, which were based on empirical trials (I-Green, Padua, Italy). The CON group did not receive any supplementation but was fed the same standardized diet as the experimental group. All owners were provided with a temperature and humidity monitoring system (Tinytag from Data Loggers, Gemini Data Loggers Ltd, West Sussex, United Kingdom), which was placed within the outdoor area. The data loggers were programmed to record temperature and humidity hourly. During each semen and blood sampling session, the data loggers were transported to the Obstetric, Gynecological, and Andrological Clinic of the Veterinary Medicine Department at the “Aldo Moro” University of Bari (Italy), where the recorded data were downloaded to calculate the Temperature-Humidity Index (THI).

**Table 2:** Phenolic composition and antioxidant activity of *Pinus taeda* Hydrolyzed Lignin^A^.

**Component**	**g/100g**
**Determined composition (g/100 g)**	
Vanillin	26.4
Eriodictyol	3.4
Quercetin	2.7
Isorhamnetin	1.6
Rosmarinic acid	1.4
Quercetin ramnoside	13.9
Methylgallate retunoside	42.3
Epigallocatechin-3-methylgallate	1.5
Ferulic acid derivates	6.7
**Antioxidant activity (**μ**mol TE**^B^ **g**^−1^ **DW**^C^**)**	
Trolox equivalent antioxidant capacity	23.9
Oxygen radical absorbance capacity	122.4

^A^According to Gerardi et al. (63) and Blando et al. (61).

^B^Trolox equivalents.

^C^Dry weight.

This dataset was employed to compute the hourly THI utilizing the formula outlined by (23):


$$\begin{array}{l} THI\;=\;\left(1.8\;\times \;AT\;+\;32\right)\;-\;\left(0.55\;-\;0.0055\;\times \;RH\right)\;\\ \;\times \;\left[\left(1.8\;\times \;AT\;+\;32\right)\;-\;58\right] \end{array}$$

where AT is the environmental temperature expressed in degrees Celsius, so that the term (1.8 × AT + 32) represents the conversion of temperature data in degrees Fahrenheit, and RH is the relative humidity as a fraction of unit.

**Figure 1:**
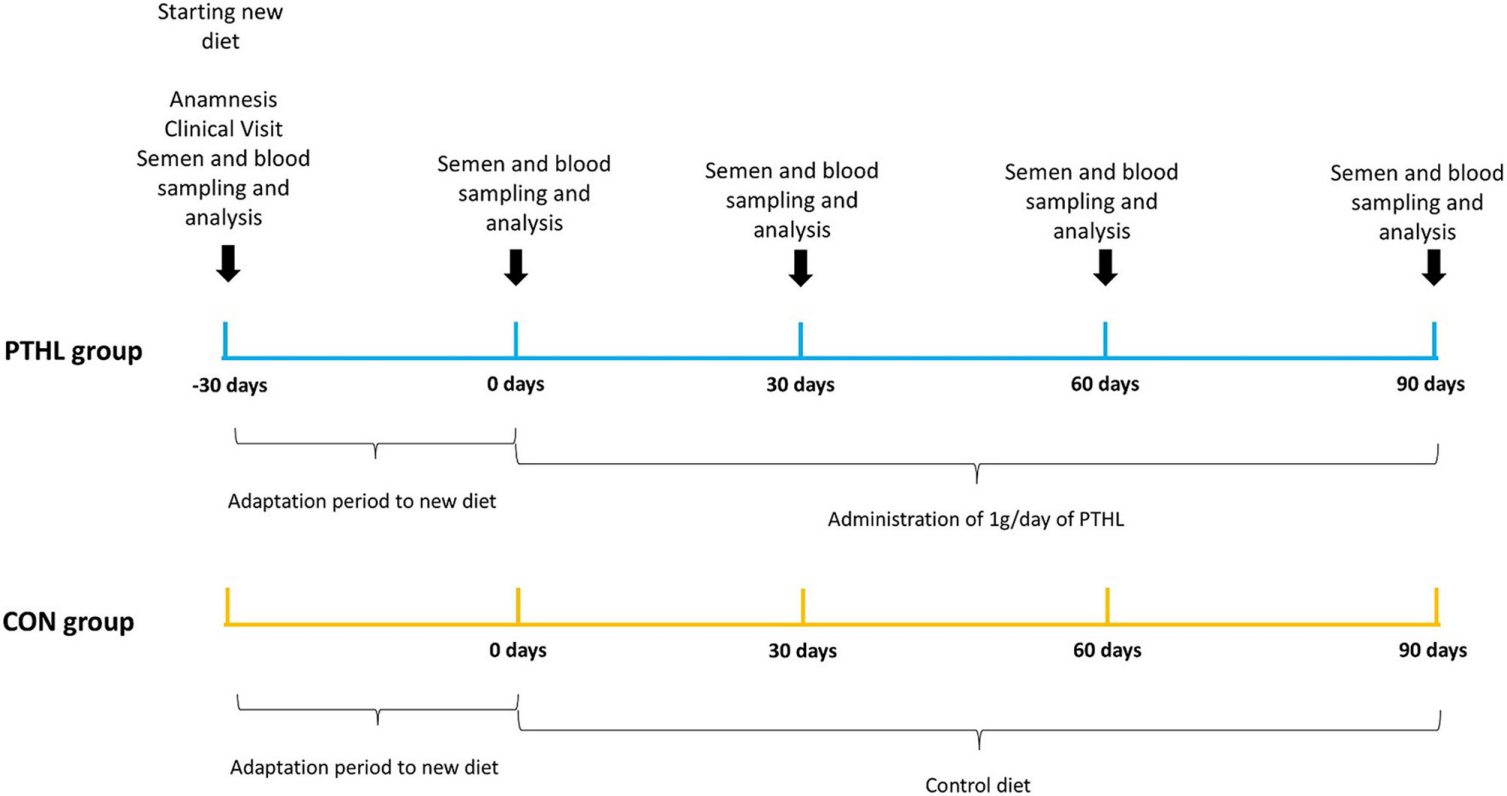
Experimental design. Timing of activities, feeding and sampling in the CON and PTHL groups during their heat stress period.

The trial lasted 120 days, after 30 days of adaptation to new diet, there was the starting time point (T0) and semen and blood samples collection were performed every 30 days (T30, T60, and T90) until the end of the trial. The experimental design is reported in Figure 1.

### 2.3 Blood samples collection and analysis

Blood samples were aseptically collected via cephalic vein puncture using disposable 22G needles and a 4 ml vacuum tube system for serum (without anticoagulant) and plasma (with 15 USP U/ml of heparin) (Becton, Dickinson Canada Inc, Vacutainer 1, Oakville, Canada). Plasma tubes were immediately centrifuged at 1,500 × g for 10 min, while serum tubes were left to clot at a refrigerated temperature for 10 min before undergoing the same centrifugation process. All plasma and serum aliquots were then stored at −80°C until analysis.

#### 2.3.1 Clinical biochemical analysis

Clinical biochemistry parameters were assessed from serum samples using an automated biochemistry analyzer (CS-300B; Dirui, Changchun, China) as described by De Palo et al. (24). The parameters analyzed included alanine aminotransferase (ALT), aspartate aminotransferase (AST), creatine phosphokinase (CPK), lactate dehydrogenase (LDH), alkaline phosphatase (ALP), glucose (Glu), blood urea nitrogen (BUN), creatinine (Crea), total serum protein (TP), albumin (Alb), cholesterol (Chol), triglycerides (Trig), non-esterified fatty acids (NEFA), calcium (Ca), phosphate (P), magnesium (Mg), and chloride (Cl) (Gesan Production Kit, Campobello di Mazara, Trapani, Italy). Additionally, globulin (Glob) levels and the albumin/globulin ratio (Alb/Glob) were derived from total protein and albumin values. Before each analytical session, the multi-parameter analyzer was calibrated using standard assay kits (Seracal, Gesan Production Kit, Campobello di Mazara, Trapani, Italy). Internal accuracy was verified using two multi-parameter control sera, a normal and a pathological one (Seracontrol N and Seracontrol P, Gesan Production Kit, Campobello di Mazara, Trapani, Italy), and was considered acceptable when deviations did not exceed 3.00% from the manufacturer's declared values. Each sample was analyzed in triplicate, with the arithmetic mean of the three readings used for statistical analysis.

#### 2.3.2 Oxidative stress assessment

Plasma samples were used to evaluate oxidative stress parameters and antioxidant enzyme activities. Thiobarbituric acid reactive substances (TBARS) were quantified spectrophotometrically following the method described by Maggiolino et al. (20), where 100 μl of plasma were mixed with a 3.7 μl/ml thiobarbituric acid solution. Reactive carbonyl derivative (RCD) levels in plasma were assessed according to Faure and Lafond (25) using the DNPH carbonyl reagent. Plasma samples (200 μl) were combined with 1 ml of water and 2 ml of 200 μl/ml trichloroacetic acid before being centrifuged at 1,000 × g for 10 min. The resulting pellet was resuspended in 1 ml of 10 mmol/L DNPH and incubated at 37.8°C for 60 min. A control sample was prepared by replacing DNPH with 1 ml of 1 mol/L hydrochloric acid. Following incubation, 1 ml of 200 μl/ml trichloroacetic acid was added, and samples were centrifuged again at 1,000 × g for 10 min. The pellet was then washed with a 1:1 ethanol-ethyl acetate solution, centrifuged, and resuspended in 1 ml of 6 mol/L guanidine (diluted in 20 mmol/L dihydrogen phosphate, pH 2.3), followed by a final incubation at 37.8°C for 40 min. Absorbance was measured at 380 nm. Hydroperoxide (Hy) levels were determined spectrophotometrically using an iodometric method described by Maggiolino et al. (26). Plasma aliquots (90 μl) were distributed into eight microcentrifuge vials (1.5 ml), with 10 μl of 10 mM TPP in methanol added to four vials to reduce ROOHs, creating a set of blank samples. The remaining four vials received 10 μl of methanol, serving as test samples. After vortexing and incubating at room temperature for 30 min, 900 μl of FOX2 reagent were added. The mixture was incubated for another 30 min before centrifugation at 2,400 × g for 10 min using a swing-out rotor (Hettich Rotenta/RP centrifuge, Hettich-Zentrifugen, Tuttlingen, Germany). The absorbance of the supernatant was recorded at 560 nm using an Ultraspec 2000 spectrophotometer (Pharmacia Biotech, Uppsala, and Sweden), and the ROOH concentration was determined based on the mean absorbance difference between blank and test samples. Protein carbonyls (PC) levels were determined spectrophotometrically as reported by Salzano et al. (27). The superoxide dismutase (SOD) (EC 1.15.1.1) activity was examined according to Misra (28), and the enzymatic activity was based on the 50% inhibition rate of epinephrine autooxidation at 480 nm (29). The SOD activity was assessed as the 50% inhibition rate of epinephrine auto-oxidation at 480 nm. The epinephrine autoxidation stimulation by traces of heavy metals present as contaminants in the reagents was prevented by adding 10–4 M EDTA in the buffer to chelate those ions. The SOD activity was expressed as U/ml. The catalase (CAT) (EC 1.11.1.6) activity was assayed by the method of Clairborne (30) as described by Tateo et al. (31). The amount of enzyme required to degrade 1 μmol of H_2_O_2_ in 60 s was defined as 1 unit of enzyme activity by following the decrease in absorbance of H_2_O_2_ at 240 nm (*e* = 40 M^−1^ cm^−1^). Its activity was expressed as U/mg of protein. The glutathione peroxidase (GPx) (EC1.11.1.9.) activity was measured according to Gunzler (32) as described by Dinardo et al. (33). The analysis was based on the measure of the rate of reduced glutathione oxidation by tertbutyl hydroperoxide, catalyzed by GPx. The constant concentration of reduced glutathione was ensured by the addition of exogenous glutathione reductase and NADPH, which converted the oxidized glutathione to reduced glutathione. The rate of oxidized glutathione formation was then measured by the change in the absorbance of NADPH at 340 nm. Its activity was expressed as nmol of NADPH oxidized/min per ml.

### 2.4 Semen collection and analysis

Semen samples were collected using an artificial vagina through manual stimulation while the dogs were exposed to swabs containing estrous bitch pheromones. The ejaculate was fractionated into three separate Falcon tubes, corresponding to the urethral, spermatic, and prostatic fractions (34, 35). All semen analyses were performed by an operator who was blind to the treatment.

#### 2.4.1 Ejaculate analysis

Ejaculate analysis was conducted following the methodology described by Alonge et al. (36). The second seminal part was analyzed by the Computer Assisted Sperm Analyzer (37), (CASA, IVOS-Sperm CASA system, Version 12.3, Hamilton Thorne, MA, USA). The CASA software (IVOS 12.3 version) was calibrated for canine semen-specific parameters, as outlined in Table 3. According to the manufacturer's instructions, each semen sample was diluted fivefold in a Tris-Fructose extender, and a 3 μl drop was placed on a Leja slide with four 20 μm chambers (Leja Products B.V., Nieuw Vennep, The Netherlands). The slide was positioned in the CASA microscope chamber, allowing the sample to stabilize before analysis. The software scanned five random, non-consecutive microscopic fields to assess semen quality. The following parameters were measured: ejaculate volume, sperm concentration, total motility, progressive motility, velocity average pathway (VAP), straight-line velocity (VSL), curvilinear velocity (VCL), amplitude of lateral head displacement (ALH), beat-cross frequency (BCF), straightness (STR), linearity (LIN), and total sperm count (TSC). VAP, expressed in μm/s, was calculated as the average velocity of a smoothed sperm path. Based on velocity thresholds, the sperm population was categorized into four subgroups: rapid spermatozoa (VAP > MVV), medium spermatozoa (LVV < VAP < MVV), slow spermatozoa (VAP < LVV), and static spermatozoa, which consisted of non-motile cells during the analysis (38).

**Table 3:** IVOS 12,3 software settings for dog semen parameters.

**Parameters**	**Cut-off value**
Frames per second (Fps)	30
Frequency	60 Hz
Temperature of analysis	37°C
Minimum contrast	75
Minimum cell size	4 pixels
Progressive cell cut-off	100 μm/s; 75% STR
Low VAP cut-off	9 μm/s
Low VSL cut-off	20 μm/s

#### 2.4.2 Oxidative stress

Prostatic fluid was collected for the determination of reactive oxygen metabolites (d-ROMs) and biological antioxidant potential (BAP). The d-ROMs were measured using a free radical elective evaluator (FREE Carpe Diem; Diacron International srl, Grosseto, Italy), which includes a spectrophotometric reader and measurement kits (d-ROMs test, Wismerll Co. Ltd., Tokyo, Japan) optimized for the FREE Carpe Diem^®^ system, following the manufacturer's protocol. For d-ROMs assessment, a 20 μl aliquot of seminal plasma was mixed with 1 ml of buffered solution (R2 kit reagent, pH 4.8) in a cuvette. Subsequently, 20 μl of chromogenic substrate (R1 kit reagent) was added. The mixture was thoroughly blended and incubated in the analyzer's thermostatic block at 37°C for 5 min, after which absorbance at 505 nm was recorded. Results were expressed in arbitrary units (U/CARR), where one unit corresponds to 0.8 mg/L of hydrogen peroxide. BAP determination was carried out using the same spectrophotometric reader and corresponding measurement kits (BAP test, Wismerll Co. Ltd., Tokyo, Japan), adhering to the manufacturer's instructions. In this assay, 50 μl of chromogenic substrate (R2 kit reagent) was combined with 1 ml of reactive solution (R1 kit reagent) in a cuvette, and absorbance at 505 nm was initially recorded. A 10 μl sample of seminal plasma was then introduced into the cuvette, followed by thorough mixing. The mixture was incubated at 37°C for 5 min in the thermostatic block of the analyzer, after which a second absorbance reading at 505 nm was obtained. Results were expressed as mmol/L of reduced ferric ions (39).

#### 2.4.3 Structural membrane integrity

As previously described in similar clinical studies (36), structural membrane integrity was assessed using the Eosin/Nigrosin staining method. The proportion of spermatozoa with an intact cytoplasmic membrane was determined by staining with Eosin/Nigrosin and analyzing at least 100 sperm cells per sample under 400 × magnification. Spermatozoa with compromised membrane integrity, including dead cells and those exhibiting structural alterations, appeared with a red or intensely pink head, whereas live sperm cells remained pale white. Since dead spermatozoa have a damaged membrane with perforations and loss of integrity, they become permeable to the dye, resulting in coloration during the vitality test. Conversely, live spermatozoa retain an intact membrane, preventing dye penetration and thus remaining unstaine (40).

#### 2.4.4 Functional membrane integrity

The functional integrity of the spermatozoa's cytoplasmic membrane was assessed using the hypo-osmotic swelling (HOS) test. In this assay, sperm cells were exposed to a hypo-osmotic solution, allowing fluid to enter the cells. The test was performed by mixing 25 μl of semen with 250 μl of a hypo-osmotic solution (containing 25 mM sodium citrate dihydrate and 75 mM D-fructose) at 37°C, followed by incubation for 30 min. After incubation, a 10 μl aliquot was placed on a microscope slide, covered with a coverslip, and examined under an optical microscope at 400 × magnification. At least 100 sperm cells were evaluated across five different fields, focusing on the presence of coiled tails. Spermatozoa with intact cytoplasmic membranes exhibited tail swelling due to water influx, whereas those with damaged membranes showed no such morphological changes (41). Sperm morphology was further analyzed, and the number of cells with naturally coiled tails was subtracted from those that exhibited coiling after incubation.

### 2.5 Data analysis

Each animal represented an experimental unit. All data sets were tested for normal distribution using the Shapiro-Wilk test and for homogeneity of variance using the Bartlett test. All parameters were analyzed using analysis of variance (ANOVA) according to the General Linear Model (GLM) procedure, following the model reported below:


(1) $$\begin{array}{c} y_{ijk}=\mu +\alpha_{i}+G_{j}+T_{k}+{\left(G\times T\right)}_{jk}+\varepsilon_{ijkl}, \end{array}$$

where *y*_*ijk*_ represents all dependent variables; μ is the overall mean; α_*i*_ is the constant of the individual dog random effect (*i* = 1,…, 40); *G* represents the effect of the *j*^th^ group (*j* = 1, 2), *T* was the effect of the *k*^th^ time (*k* = 1, …, 4), *G* × *T* represents the binary interaction between the *j*^th^ group and the *k*^th^ time (1, …, 8). Significance was set at *P* < 0.05, and the results were expressed as predicted means and mean standard errors. All the analysis was performed using SAS software (42).

## 3 Results

### 3.1 Clinical biochemical results

**Figure 2:**
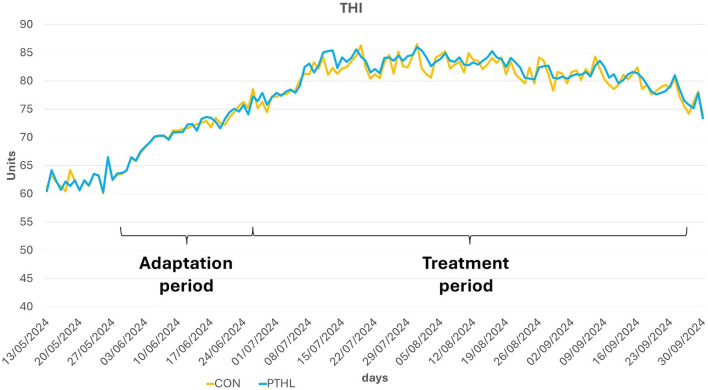
Average maximum THI registered for the two experimental groups during the trial.

Average maximum THI values registered for each experimental group were reported in Figure 2. All biochemical parameters of all dogs were in the reference value (62). The fixed effects of group, time and their interaction on serum biochemical-clinical profile are shown in Table 4. Total protein, Albumin, Glcemia, ALT, AST, ALP (*P* < 0.01), and Urea (*P* < 0.05) were affected by the interaction of group and time. Total protein levels decreased contantly until 60 days in CON group (*P* < 0.01) and then remained constant, while in PTHL group showed at 90 days lower values then 0 days (*P* < 0.01). Moreover, PTHL group reported higher serum calues than CON group at 60 and 90 days of trial (*P* < 0.01). Albumin serum levels decreased in CON group after 60 days (*P* < 0.01) remaining constant until 90, while decreased at 90 days in PTHL group (*P* < 0.05), showing no differences between groups within each experimental time (*P* > 0.05). The Urea level decreased at 60 days in CON group and at 90 days in PTHL group (*P* < 0.01), reporting higher values in PTHl grop at 60 and 90 days (*P* < 0.05). Clycemia decreased in CON group at 30 days (*P* < 0.01) and then again at 90 days (*P* < 0.01), while decreased directly at 90 days in PTHL grooup (*P* < 0.01), showing in this last group higher values than CON one from 30 to 90 days of trial (*P* < 0.01). ALT serum levels inreased at 30 and 60 days in CON group (*P* < 0.01), while increased at 60 days in PTHL one (*P* < 0.01), reporting in this last one costantly lower values than CON group from 30 to 90 days (*P* < 0.01). Differently, AST increased at 30 days in CON group and at 60 days in PTHL group (*P* < 0.01), remaining constant until 90 days in both groups but showing higher values in CON one from 30 to 90 days (*P* < 0.01). ALP levels increased at 60 days in CON group and at 90 days in PTHL one (*P* < 0.01), with no differences between groups within each experimental time (*P* > 0.05). The fixed effects of group, time and their interaction on chloride, sodium, potassium, magnesium, phosphorous and calcium serum concentration are presented in Table 5. Chloride was affected by the interaction of group and treatment. After 90 days it showed lower values than 0 and 30 days in CON group (*P* < 0.01) and than PTHL group at the sema time (*P* < 0.01).

**Table 4:** Total protein, albumin, urea, uric acid, creatinine, bilirubin, triglyceride, glycemia, alanine amino transferase (ALT), aspartate amino transferase (AST), alkaline phosphatase (ALP) and cholesterol serum concentration in heat-stressed dogs over 90 days, with (PTHL) or without (CON) dietary supplementation of *Pinus taeda* hydrolyzed lignin.

**Parameter**	**Group**	**Time (days)**				**SEM^1^**	***F and P-value***			**Reference value^4^**
		**0**	**30**	**60**	**90**		**G** ^2^	**T** ^3^	**G** × **T**	
Total protein (g/dL)	CON	6.78^A^	6.02^B^	5.42^C, X^	5.22^C, X^	0.27	*F* = 20.1 < 0.001	*F* = 32.6 < 0.001	*F* = 9.8 < 0.001	5.4–7.1
	PTHL	6.65^A^	6.17	6.12^Y^	6.02^B, Y^					
Albumin (g/dL)	CON	3.08^A^	2.71	2.26^B^	2.18^B^	0.12	*F* = 0.18 0.671	*F* = 19.7 < 0.001	*F* = 8.4 < 0.001	2.6–3.3
	PTHL	3.11^a^	3.06	2.96	2.68^b^					
Urea (mmol/L)	CON	58.73^A^	55.42^A^	43.17^B, x^	40.57^B, x^	8.71	*F* = 15.4 < 0.001	*F* = 14.6 < 0.001	*F* = 3.4 0.021	21–60
	PTHL	57.73^A^	56.49	50.47^y^	46.91^B, y^					
Uric acid (mg/dL)	CON	0.65	0.66	0.84	0.89	0.12	*F* = 1.5 0.236	*F* = 1.3 0.302	*F* = 0.9 0.434	0.0–2.0
	PTHL	0.69	0.65	0.71	0.73					
Creatinine (mg/dL)	CON	1.36	1.32	1.28	1.24	0.11	*F* = 0.9 0.347	*F* = 1.5 0.228	*F* = 0.6 0.621	0.5–1.5
	PTHL	1.39	1.25	1.31	1.29					
Bilirubine (mg/dL)	CON	0.29	0.23	0.30	0.28	0.06	*F* = 0.4 0.516	*F* = 0.2 0.675	*F* = 0.1 0.801	0.1–0.5
	PTHL	0.27	0.29	0.26	0.34					
Triglyceride (mg/dL)	CON	34.35	33.67	31.38	31.34	9.105	*F* = 1.1 0.337	*F* = 1.8 0.179	*F* = 0.6 0.542	30.1–38.1
	PTHL	35.93	34.35	33.52	33.32					
Glycemia (mg/dL)	CON	99.05^A^	81.29^B, X^	71.69^BC, X^	66.79^C, X^	5.56	*F* = 19.9 < 0.001	*F* = 33.7 < 0.001	*F* = 12.5 < 0.001	65–118
	PTHL	101.83^A^	98.14^Y^	89.44^Y^	86.12^B, Y^					
ALT^5^ (IU/L)	CON	32.25^A^	49.93^B, X^	65.73^C, X^	67.18^C, X^	6.12	*F* = 25.2 < 0.001	*F* = 29.4 < 0.001	*F* = 10.3 < 0.001	21–102
	PTHL	34.68^A^	36.23^Y^	44.99^B, Y^	44.67^B, Y^					
AST^6^ (IU/L)	CON	23.36^A^	41.15^B, X^	46.53^B, X^	47.68^B, X^	3.34	*F* = 22.1 < 0.001	*F* = 28.6 < 0.001	*F* = 11.2 < 0.001	0.0–66.0
	PTHL	22.25^A^	26.77^Y^	31.91^B, Y^	33.67^B, Y^					
ALP^7^ (IU/L)	CON	23.62^A^	29.87	34.22^B^	37.34^B^	3.61	*F* = 1.8 0.178	*F* = 15.7 < 0.001	*F* = 3.9 0.008	20–156
	PTHL	24.06^A^	26.45	28.71	32.53^B^					
Cholesterol (mg/dL)	CON	178.93	179.37	203.14	197.87	22.33	*F* = 0.2 0.689	*F* = 0.9 0.442	*F* = 0.7 0.603	135–270
	PTHL	170.4	180.76	186.12	194.66					

^1^Standard error of the means.

^2^Group.

^3^Time.

^4^Kaneko et al. (62).

^5^Alanine aminotransferase.

^6^Aspartate aminotransferase.

^7^Alkaline phosphatase.

Different letters on the same parameter of the same group show statistical differences: A, B, C = *P* < 0.01. Different letters at the same time for the same parameter show statistical differences: X, Y = *P* < 0.01; x, y = *P* < 0.05.

**Table 5:** Chloride, sodium, potassium, magnesium, phosphorus and calcium serum concentration in heat-stressed dogs over 90 days, with (PTHL) or without (CON) dietary supplementation of *Pinus taeda* hydrolyzed lignin.

**Parameter**	**Group**	**Time (days)**				**SEM^1^**	***P-value***			**Reference value^4^**
		**0**	**30**	**60**	**90**		**G** ^2^	**T** ^3^	**G** × **T**	
Chloride (mEq/L)	CON	113.82^A^	112.63^A^	98.71	92.15^B, X^	10.91	*F* = 20.5 < 0.001	*F* = 28.7 < 0.001	*F* = 9.2 < 0.001	105.0–115.0
	PTHL	113.47	110.85	111.63	113.23^Y^					
Sodium (mmol/L)	CON	144.72	149.79	145.99	142.52	13.88	*F* = 0.16 0.689	*F* = 0.13 0.912	*F* = 0.57 0.591	141.0–152.0
	PTHL	147.47	146.17	144.95	145.83					
Potassium (mg/dL)	CON	4.48	4.46	4.51	4.51	0.35	*F* = 0.06 0.819	*F* = 0.43 0.711	*F* = 0.69 0.507	4.4–5.3
	PTHL	4.52	4.49	4.53	4.55					
Magnesium (mg/dL)	CON	2.16	2.08	2.13	2.11	0.22	*F* = 0.04 0.840	*F* = 0.35 0.765	*F* = 0.66 0.554	1.8–2.4
	PTHL	2.26	2.22	2.14	2.24					
Phosphorus (mg/dL)	CON	4.32	4.66	4.41	4.78	0.48	*F* = 0.09 0.771	*F* = 0.44 0.697	*F* = 0.23 0.817	2.6–6.2
	PTHL	4.03	4.27	4.33	4.29					
Calcium (mg/dL)	CON	10.55	10.65	11.07	10.82	1.29	*F* = 0.96 0.336	*F* = 0.41 0.677	*F* = 0.52 0.752	9–12
	PTHL	10.43	9.95	10.31	10.27					

^1^Standard error of the means.

^2^Group.

^3^Time.

^4^Kaneko et al. (62).

Different letters on the same parameter of the same group show statistical differences: A, B = *P* < 0.01. Different letters at the same time for the same parameter show statistical differences: X, Y = *P* < 0.01.

### 3.2 Oxidative stress results

The fixed effects of group, time and their interaction on the oxidative profile are shown in Table 6. FRAP, SOD, CAT and GSPx were affected by the interaction of group and treatment (*P* < 0.01), TBARs were affected by time (*P* < 0.05). TBARs plasma levels increased in at 30 days in CON group (*P* < 0.01) and then remained constant until 90 days, while in PTHL group TBARs plasma levels at 90 days were higher than 0 days (*P* < 0.05). At 30, 60 (*P* < 0.05) and 90 (*P* < 0.01) days, CON group showed higher TBARs levels than PTHL one. FRAP plasma levels increased in PTHL group at 30 days and then at 90 days (*P* < 0.01), reporting higher values than CON group from 30 to 90 days of trial (*P* < 0.01). SOD plasma levels at 30 days decreased in CON group and increased in PTHL group (*P* < 0.01), showing lower values in CON group from 30 to 90 days of trial (*P* < 0.01). The CAT levels increased at 60 and then at 90 days in CON group (*P* < 0.01), while in PTHL group shoved higher values at 90 days compared to 0 days (*P* < 0.01). Moreover, the PL grop showed higher CAT values at 60 and 90 days (*P* < 0.01). The GSPx plasma levels decreased in CON group at 60 days, reporting lower values tha PTHL group at 60 and 90 days (*P* < 0.01).

**Table 6:** Thiobarbituric acid reactive substances (TBARs), hydroperoxides, protein carbonyls, Ferric Reducing Antioxidant Power (FRAP), superoxide dismutase (SOD), catalase (CAT) and glutathione peroxidase (GSPx) serum concentration in heat-stressed dogs over 90 days, with (PTHL) or without (CON) dietary supplementation of *Pinus taeda* hydrolyzed lignin.

**Parameter**	**Group**	**Time (days)**				**SEM^1^**	***P-value***		
		**0**	**30**	**60**	**90**		**G** ^2^	**T** ^3^	**G** × **T**
TBARs ^4^ (mmol/mL)	CON	1.29^A^	2.36^B, x^	2.85^B, x^	3.09^B, X^	0.18	*F* = 1.5 0.223	*F* = 3.1 0.042	*F* = 0.5 0.622
	PTHL	1.33^a^	1.68^y^	1.66^y^	1.82^b, Y^				
Hydroperoxides (mmol/mL)	CON	5.15	5.23	5.17	5.34	0.51	*F* = 0.2 0.671	*F* = 1.0 0.369	*F* = 0.1 0.706
	PTHL	4.98	5.09	4.84	4.91				
Protein carbonyls (μmol/mg Protein)	CON	93.88	89.23	92.94	88.01	10.18	*F* = 0.6 0.452	*F* = 0.6 0.662	*F* = 0.5 0.467
	PTHL	94.03	90.56	89.78	93.21				
FRAP^5^ (μmol TE/mL)	CON	47.64	45.92^X^	46.27^X^	42.35^X^	0.41	*F* = 50.2 < .0001	*F* = 55.3 < .0001	*F* = 48.7 < .0001
	PTHL	46.01^A^	58.25^B, Y^	58.93^B, Y^	61.53^C, Y^				
SOD^6^ (U/mL)	CON	44.39^A^	36.81^B, X^	32.20^B, X^	34.01^B, X^	1.140	*F* = 45.8 < .0001	*F* = 60.1 < .0001	*F* = 40.3 < .0001
	PTHL	43.98^A^	49.72^B, Y^	51.31^B, Y^	50.57^B, Y^				
CAT ^7^ (U/mL)	CON	2.24^A^	2.18^AB^	1.71^B, X^	1.55^C, X^	0.07	*F* = 30.5 < .0001	*F* = 25.7 < .0001	*F* = 28.4 < .0001
	PTHL	2.26^A^	2.38	2.41^Y^	2.70^B, Y^				
GSPx ^8^ (nmol NADPH ox/mL)	CON	2.41^A^	2.33	1.80^B, X^	1.71^B, X^	0.09	*F* = 33.2 < .0001	*F* = 42.5 < .0001	*F* = 35.6 < .0001
	PTHL	2.32	2.43	2.49^Y^	3.52^Y^				

^1^Standard error of the means.

^2^Group.

^3^Time.

^4^TBARS - Thiobarbituric Acid Reactive Substances.

^5^FRAP - Ferric Reducing Antioxidant Power.

^6^SOD - Superoxide Dismutase.

^7^CAT - Catalase.

^8^GSPx - Glutathione Peroxidase.

Different letters on the same row show statistical differences during time in the same group: A, B, C, D = *P* < 0.01; Different letters on the same column show statistical differences between groups at the same aging time: X, Y = *P* < 0.01.

### 3.3 Semen results

**Figure 3:**
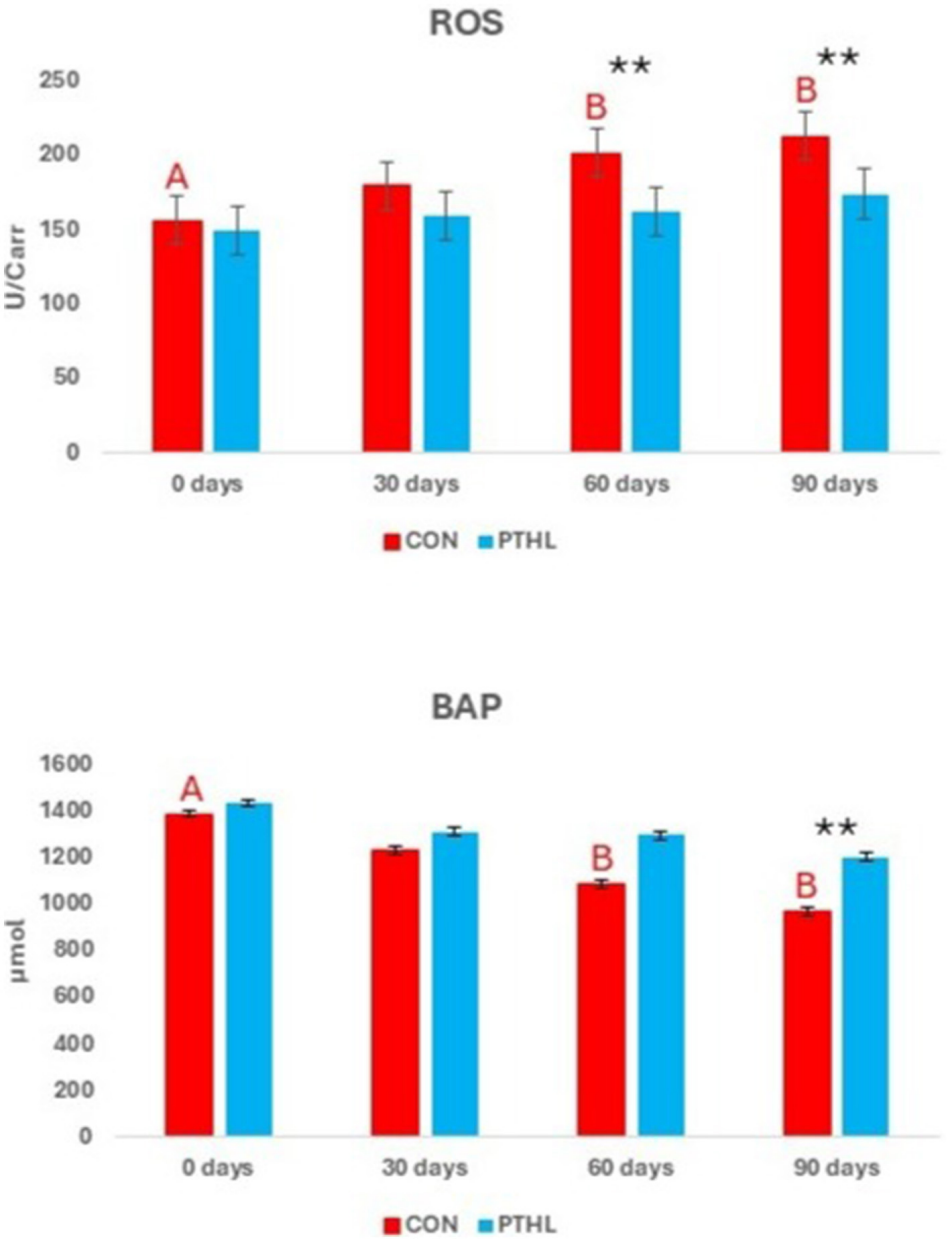
Reactive oxygen metabolites (d-ROMs) and biological antioxidant potential (BAP) concentrations in canine seminal plasma (third fraction). Different letters of the same colors show statistical differences among times in the same group: A, B = *P* < 0.01; ^**^ = *P* < 0.01 showed statistical differences between groups in the same experimental time.

The fixed effects of group, time and their interaction on sperm parameters are shown in Table 7. Volume, concentration, TSN, STR, LIN, HOS (*P* < 0.01), VCL, and Sperm viability (*P* < 0.05) where affected by the interaction of group and time. Semen volume decreased at day 90 compared to day 0 in both CON (*P* < 0.01) and PTHL (*P* < 0.05) groups, with higher values observed in the latter (*P* < 0.05). Sperm concentration decreased at 30 (*P* < 0.05), 60 and 90 days in CON group (*P* < 0.01) and at 60 days in PTHL group (*P* < 0.01), showing in this last one higher values at 60 and 90 days (*P* < 0.01). The TSN decreased at 30 (*P* < 0.05), 60 and 90 (*P* < 0.01) days in CON group, while only at 60 days in PTHL one (*P* < 0.01), showing in this last group higher values at 60 and 90 days compared to CON group (*P* < 0.01). VCL values decreased in CON group at 30 days (*P* < 0.05) and from 30 to 90 days remained lower than PTHL group (*P* < 0.05). STR values of CON group decreased at 60 days (*P* < 0.01), showing lower values tha PTHL group at 60 (*P* < 0.05) and 90 (*P* < 0.01) days. LIN and HOS values of CON group at 90 days were lower than 0 days (*P* < 0.01) and HOS showed lower values at 90 days than PTHL group (*P* < 0.01). Also sperm viability showed lower values at 90 days compared to 0 days in CON group (*P* < 0.05). The fixed effects of group, time and their interaction on spermatozoa motility parameters are reported in Table 8. Total motility, progressive motility, rapid, slow and static values where affected by the interaction of group and time (*P* < 0.01). Both total and progressive sperm motility in CON group decreased at 60 days compared to day 0 (*P* < 0.01), with a further decline at 90 days (*P* < 0.01). Additionally, at 90 days, CON group revealed lower total motility (*P* < 0.01), and at both 60 (*P* < 0.05) and 90 (*P* < 0.01) days, lower progressive motility compared to PTHL group. The percentage of rapid sperm movements decreased at 30 and 60 days in CON dogs (*P* < 0.01) and at day 90 in PTHL dogs (*P* < 0.01) compared to their respective day 0 values. Additionally, the percentage of rapid movements was higher in PTHL group at 60 and 90 days (*P* < 0.01). The percentage of slow movements increased in both groups from 30 to 90 days (*P* < 0.01) compared to 0 days (*P* < 0.01), but it was higher in CON group than in PTHL one from 30 to 90 days (*P* < 0.01). Moreover, the percentage of static movements increased only in CON dogs starting from 30 days compared to 0 days (*P* < 0.01) and was higher than in PTHL dogs from 30 to 90 days (*P* < 0.01). Figure 3 illustrates the concentrations of d-ROMs and BAP in the seminal plasma. d-ROMs levels increased only in the seminal plasma of CON group at 60 and 90 days compared to 0 days (*P* < 0.01), and at both time points, their concentrations were higher than those in PTHL group (*P* < 0.01). In contrast, BAP levels in the seminal plasma of CON group decreased at 60 and 90 days compared to 0 days (*P* < 0.01), and at 90 days, they were lower than those in PTHL group (*P* < 0.01).

**Table 7:** Volume, concentration, total sperm number (TSN), velocity average pathway (VAP), straight line velocity (VLS), curvilinear velocity (VCL), amplitude of lateral head displacement (ALH), beat-cross frequency (BCF), straightness (STR), linearity (LIN), hypo-osmotic swelling test (HOS) and sperm viability of semen in heat-stressed dogs over 90 days, with (PTHL) or without (CON) dietary supplementation of *Pinus taeda* hydrolyzed lignin.

**Parameter**	**Group**	**Time (days)**				**SEM^1^**	***P-value***		
		**0**	**30**	**60**	**90**		**G** ^2^	**T** ^3^	**G** × **T**
Volume (mL)	CON	3.35^A^	3.12	2.78	2.37^B, x^	0.26	*F* = 4.2 0.045	*F* = 4.6 0.0085	*F* = 6.1 0.0003
	PTHL	3.33^a^	3.16	3.02	2.96^b, y^				
Concentration (M/mL)	CON	254.41^Aa^	232.50^Ab^	206.32^B, x^	180.16^B, x^	18.35	*F* = 6.2 0.0201	*F* = 30.5 < .0001	*F* = 10.5 0.0013
	PTHL	248.33^a^	236.66	223.22^by^	226.21^b, y^				
TSN (M)	CON	869.34^Aa^	736.11^Ab^	584.22^B, X^	448.09^C, X^	52.85	*F* = 25.3 < .0001	*F* = 40.2 < .0001	*F* = 20.7 < .0001
	PTHL	854.49^A^	751.99	668.07^B, Y^	658.44^B, Y^				
VAP (μm/s)	CON	96.31	88.44	84.22	81.16	10.35	*F* = 0.2 0.6652	*F* = 0.8 0.4701	*F* = 0.5 0.6229
	PTHL	95.33	92.15	93.62	88.61				
VLS (μm/s)	CON	95.18	90.66	90.14	88.22	11.25	*F* = 0.3 0.6225	*F* = 0.7 0.3328	*F* = 0.4 0.5383
	PTHL	96.41	93.45	94.19	92.77				
VCL (μm/s)	CON	173.33^a^	156.71^b, x^	154.40^b, x^	155.19^bx^	13.88	*F* = 5.3 0.0257	*F* = 3.1 0.0446	*F* = 3.7 0.0184
	PTHL	178.16	173.36^y^	170.17^y^	169.09^y^				
ALH (μ)	CON	5.22	5.54	5.72	5.59	0.84	*F* = 0.5 0.7745	*F* = 0.6 0.5769	*F* = 0.3 0.6106
	PTHL	5.71	5.88	5.49	6.01				
BCF (Hz)	CON	24.36	25.12	23.49	24.81	3.62	*F* = 0.6 0.5913	*F* = 0.4 0.5116	*F* = 0.7 0.4289
	PTHL	26.31	26.86	25.37	26.21				
STR (VSL/VAP)	CON	84.81^A^	79.21	76.19^Bx^	74.88^BX^	7.33	*F* = 30.1 < .0001	*F* = 35.7 < .0001	*F* = 28.4 < .0001
	PTHL	85.12	82.53	83.16^y^	81.41^Y^				
LIN (VSL/VCL)	CON	59.31^A^	56.82	53.21	49.83^BX^	4.18	*F* = 9.5 0.0030	*F* = 12.8 0.0002	*F* = 15.2 < .0001
	PTHL	60.66	59.33	56.61	57.47^Y^				
HOS (% cureled)	CON	94.33^A^	90.44	88.61	85.95^B^	6.31	*F* = 1.5 0.2214	*F* = 7.1 0.0007	*F* = 6.8 0.0004
	PTHL	93.66	91.93	90.21	89.89				
Sperm viability (Eo-Nig) (%)	CON	92.49^a^	90.12	88.26	86.66^b^	4.49	*F* = 0.2 0.6443	*F* = 1.1 0.3221	*F* = 5.9 0.0159
	PTHL	93.01	92.12	91.44	90.40				

^1^Standard error of the means.

^2^Group × Time.

^3^G is group and T is time.

Different letters on the same line show statistical differences during time in the same group: A, B = *P* < 0.01; a, b = *P* < 0.05; Different letters on the same row show statistical differences between groups at the same aging time: X, Y = *P* < 0.01; x, y = *P* < 0.05.

**Table 8:** Total motility, progressive motility, and the distribution of rapid, medium, slow, and static movements of spermatozoa in heat-stressed dogs over 90 days, with (PTHL) or without (CON) dietary supplementation of *Pinus taeda* hydrolyzed lignin.

**Parameter**	**Group**	**Time (days)**				**SEM^1^**	***P*** **-value**		
		**0**	**30**	**60**	**90**		**Group**	**Time**	**G** × **T**^2^
Total motility (%)	CON	91.82^A^	87.87^AB^	82.41^B^	76.19^CX^	7.98	*F* = 50.4 < .0001	*F* = 45.7 < .0001	*F* = 38.2 < .0001
	PTHL	89.66	88.33	86.66	87.66^Y^				
Progressive motility (%)	CON	71.37^A^	69.44^A^	63.04^Bx^	57.56^CX^	6.12	*F* = 5.0 0.0315	*F* = 40.9 < .0001	*F* = 35.6 < .0001
	PTHL	72.22	70.33	68.52^y^	67.91^Y^				
Rapid (%)	CON	91.26^A^	85.55^B^	72.21^C, X^	67.18^C, X^	5.58	*F* = 60.2 < .0001	*F* = 55.1 < .0001	*F* = 45.9 < .0001
	PTHL	90.33^A^	88.64	85.66^Y^	81.51^B, Y^				
Medium (%)	CON	9.47	9.33	10.19	9.89	1.17	*F* = 0.3 0.4401	*F* = 0.4 0.5992	*F* = 0.6 0.6914
	PTHL	9.88	10.28	9.29	9.46				
Slow (%)	CON	4.42^A^	15.12^B, X^	16.28^B, X^	17.62^B, X^	0.43	*F* = 50.3 < .0001	*F* = 48.7 < .0001	*F* = 42.2 < .0001
	PTHL	5.02^A^	9.19^B, Y^	10.31^B, Y^	10.49^B, Y^				
Static (%)	CON	2.41^A^	4.83^B, X^	5.61^B, X^	5.44^B, X^	0.44	*F* = 10.6 0.0015	*F* = 12.3 0.0002	*F* = 20.4 < .0001
	PTHL	2.04	2.64^Y^	2.82^Y^	2.70^Y^				

^1^Standard error of the means.

^2^Group × Time.

Different letters in the same row show statistical differences during time in the same group: A, B = *P* < 0.01; a, b = *P* < 0.05; Different letters on the same column show statistical differences between groups at the same aging time: X, Y = *P* < 0.01; x, y = *P* < 0.05.

## 4 Discussion

This study evaluated the efficacy of PTHL dietary supplementation in mitigating oxidative stress and preserving sperm quality in dogs subjected to heat stress conditions. While prior study demonstrated the benefits of PTHL in enhancing antioxidant status and sperm quality in clinically healthy dogs (17), this research extended the inquiry to heat stress scenarios, yielding new insights into its protective effects under adverse environmental conditions. This study provides novel evidence that dietary PTHL supplementation can attenuate the physiological and reproductive impacts of heat stress, supporting its potential application in canine breeding programs. Environmental stressors, including heat, significantly affect hematological parameters in vertebrates, often signaling stress conditions (43). Blood, as a dynamic biomarker, revealed notable alterations in this study. Specifically, reductions in serum total protein, albumin, urea, and glucose levels, coupled with increases in ALT, AST, and ALP levels, highlighted the physiological strain imposed by heat stress. These changes, observed in both CON and PTHL groups, appeared later and were less pronounced in PTHL-supplemented dogs, underscoring the potential mitigating role of PTHL. The reduction in total protein and albumin levels in heat-stressed dogs is consistent with previous findings (4, 44, 45). These changes likely reflect cellular damage, coupled with increased metabolic activity and oxygen consumption, which together can trigger widespread cellular necrosis through protein denaturation (46). Concurrently, elevated AST and ALT levels point to liver dysfunction, which aligns with prior research suggesting impaired hepatic blood flow and oxygen delivery during thermal stress (45). The observed hypoglycemia likely arises from the dual impact of increased glucose utilization to meet thermoregulatory demands and enhanced insulin absorption due to peripheral vasodilation (47, 48). Alterations in serum urea and chloride concentrations under heat stress could be attributed to compensatory mechanisms for maintaining hydration. Excessive water intake driven by heat exposure often results in overhydration, leading to alterations in these parameters (49). Heat stress is a well-documented trigger for oxidative stress, disrupting the balance between the production of ROS and the antioxidant defense mechanisms (1). Oxidative stress induced by heat stress is well-documented in several species (50–52). ROS, such as superoxide anions, hydrogen peroxide, and hydroxyl radicals, are natural by-products of aerobic metabolism with dual roles. On the one hand, they contribute to essential processes such as immune defense and intracellular signaling. On the other hand, excessive ROS accumulation causes oxidative damage by reacting with cellular macromolecules, including lipids, proteins, and DNA. This damage occurs when the rate of ROS production surpasses the neutralizing capacity of endogenous antioxidants, which include enzymatic (e.g., SOD, CAT) and non-enzymatic (e.g., glutathione, vitamins C and E) components (1). Although research on the effects of heat stress in dogs is limited, Burgio, Forte, Prete, Maggiolino, De Palo, Aiudi, Rizzo, Carbonari, Lacalandra and Cicirelli (4) provided evidence supporting the impact of heat stress on oxidative stress markers in this species. Our findings confirm these effects, demonstrating increased TBARS and ROS levels while antioxidant enzyme activity was reduced. Specifically, exposure to heat stress led to a marked imbalance in redox homeostasis: plasma TBARS and ROS levels were significantly elevated, while the activities of key antioxidant defenses—SOD, CAT and GSPx—and overall antioxidant capacity (as measured by FRAP) were significantly suppressed compared to dogs maintained under thermoneutral conditions. Conversely, dietary antioxidant supplementation has been shown to enhance the bioavailability of polyphenolic and other bioactive compounds in target tissues, resulting in a robust, measurable increase in total antioxidant capacity across multiple organ systems (53). This effect is particularly relevant for polyphenolic compounds found in plants, which possess potent antioxidant properties (54). Our study extends previous research by showing that PTHL supplementation not only enhances antioxidant status in healthy dogs but also plays a protective role under heat stress conditions. However, considering the strong connection between heat stress and oxidative stress, such dietary interventions could offer a promising approach to alleviate the negative effects of heat stress by enhancing the antioxidant defense system and reducing oxidative damage (54, 55). Our findings reveal a significant increase in plasma TBARS levels of CON dogs as early as 30 days into the trial. In contrast, PTHL dogs showed a delayed rise in TBARS levels, which became apparent only at 90 days and remained notably lower than those observed in the CON group. These results suggest that PTHL supplementation not only mitigated the extent of lipid oxidation but also effectively delayed its onset. Moreover, the concentrations of antioxidant enzymes, including SOD, CAT, and GSPx, were consistently higher in PTHL group compared to CON dogs. Additionally, the plasma of PTHL dogs showed significantly higher FRAP values than that of CON dogs. Together, these findings highlight the potential of PTHL supplementation to fortify the plasma's antioxidant defense system, providing effective protection against the oxidative challenges imposed by heat stress.

Heat stress negatively affects not only the oxidative status but also sperm quality of male dogs, reducing reproductive performance. In dogs, elevated temperatures are associated with reduced libido, impaired spermatogenesis, lower sperm concentration, poor sperm quality, decreased testicular weight, and a temporary period of partial or total infertility (54, 55). Sperm parameters, including motility and vigor, may be negatively impacted, while morphological defects such as acrosome degradation, proximal cytoplasmic droplets, bent tails at the head, small heads, and isolated heads become more prevalent, as observed in bulls (56). In this study, heat stress worsened the semen characteristics. In both groups, before the warmer months (T0), the parameters examined (volume, motility, vigor, sperm concentration, total sperm count and morphology) were within the normal range. Afterwards, with an increase in THI (in July and August), the semen characteristics have worsened. Volume, concentration and TSN progressively decreased during the study, as did the motility parameters. The volume decreased in both groups, reaching statistically significant differences at T90. This reduction was more evident in the CON group than in the PTHL group. Concentration and TSN, instead, were already reduced at T30 in the CON group, while in the PTHL group this occurred at T60. At T90, both parameters were worse in the CON group than in the PTHL group. Probably, these negative effects are due to ROS produced by the animals' exposure to high temperatures, which negatively affect spermatogenesis and can cause cellular damage and apoptosis (57). This hypothesis is also supported by the results of sperm viability, where a reduction in live spermatozoa was observed in the control animals compared to the treated group at T90. Spermatozoa viability worsened significantly only in the CON group, while the PTHL group did not show any significant decline. However, in the PTHL group, the seminal parameters worsened less during the summer months than in the CON group. This is likely due to the antioxidant effect of a polyphenolic mixture of substances derived from the hydroxylation of *Pinus taeda* lignin. Specifically, PTHL supplementation can significantly increase semen volume and sperm motility, as demonstrated in healthy dogs under normal environmental conditions and temperatures (17). Furthermore, in this study, total motility decreased in both groups but at different times: at T30 in the CON group and at T90 in the PTHL group. In the PTHL group, progressive motility and the percentage of rapidly moving sperm did not worsen significantly, and the proportion of slow and static sperm did not increase as observed in the control group in August. This was happening probably on the antioxidant power of polyphenols contained in PTHL, which can improve sperm quality at the beginning of the sperm differentiation and development. Itis well known that oxidative stress is one of the major issues associated with sperm function (58) and sperm motility is one of the factors limiting male fertility. In fact, spermatozoa are sensitive to oxidative stress because they are well endowed with polyunsaturated fatty acids, which are vulnerable to free radical attack at the alpha methylene carbons adjacent to the carbon–carbon double bonds. In addition, the spermatozoa's capacity for antioxidative defense is relatively low/vulnerable in comparison to other cells/tissues that are susceptible (59). Furthermore, spermatozoa are attacked by the oxidative effect of leukocytes, such as neutrophils, which are present in the male genitals for infection and other causes. The literature shows that antioxidants may play a primary role in protecting male germ cells against oxidative action (60). Our results encourage considering the alimentary approach of balanced food supplementation for the solution of dog subfertility. Balanced feed supplementation and integration could mitigate the negative impact of heat stress in canine breeding.

## 5 Conclusions

This study provides compelling evidence supporting the efficacy of PTHL dietary supplementation in mitigating the detrimental effects of heat stress on oxidative status and sperm quality in dogs. PTHL supplementation effectively delayed and reduced lipid peroxidation while enhancing enzymatic antioxidant activity (SOD, CAT, and GSPx) and FRAP. Furthermore, it preserved seminal parameters such as progressive motility and the percentage of rapid-movement sperm, which were significantly compromised in CON group. Incorporating dietary supplementation into breeding programs, PTHL presents a promising natural strategy to alleviate the negative impacts of heat stress, safeguard reproductive health, and improve fertility outcomes in dogs. In light of these findings, PTHL dietary supplementation proves to be a natural, sustainable and effective strategy to strengthen the antioxidant defenses of dogs, enhance their overall well being, and safeguard sperm quality, even under the challenging conditions of heat stress. While our findings highlight the benefits of PTHL supplementation, certain limitations must be acknowledged. Variations in individual responses to heat stress and supplementation could not be fully accounted for. Additionally, the study duration may not reflect the cumulative effects of prolonged heat stress exposure. Future research should investigate different dosages, longer supplementation periods, and potential interactions with other dietary antioxidants to optimize reproductive performance in heat-stressed dogs.

## Data Availability

The raw data supporting the conclusions of this article will be made available by the authors, without undue reservation.
